# How to Enable Participation until the End of Life? A Survey of German Occupational Therapists Working in Palliative Care

**DOI:** 10.3390/jcm12165257

**Published:** 2023-08-12

**Authors:** Christian Volberg, Pauline Fleck, Paula Vradelis, Astrid Morin, Martin Gschnell, Anna Elisabeth Pape

**Affiliations:** 1 Department of Anesthesiology & Intensive Care Medicine, Faculty of Medicine, Philipps University of Marburg, 35032 Marburg, Germany; 2 Research Group Medical Ethics, Faculty of Medicine, Philipps University of Marburg, 35032 Marburg, Germany; 3 Department of Dermatology and Allergology, Faculty of Medicine, Philipps University of Marburg, 35032 Marburg, Germany; 4 Department of Therapy Somatic, Klinikum Bremen-Ost, 28325 Bremen, Germany

**Keywords:** occupational therapy, palliative care, German palliative medicine, end-of-life-care, participation, survey

## Abstract

Background: In palliative care, the needs of people with life-limiting illnesses must be addressed with the support of a multidisciplinary team. Occupational therapy is a profession that focuses on activity and participation. In Germany, there are no surveys to date that demonstrate the work of occupational therapists in palliative care and which problems can occur in this field. Aim: The aim of this study is to describe the work and problems of occupational therapists in German palliative care. Design: The survey consists of 17 questions and could be conducted anonymously. Descriptive statistics and a thematic analysis of the free text responses were used to analyze data. Setting/participants: Respondents were German occupational therapists who are members of the “German Association of Occupational Therapy”. Results: A total of 281 valid responses were evaluated, of which 120 respondents work in the context of palliative care. Most of them provide needs-based therapies (74%), train relatives (69%), or help patients with positioning (69%) or relaxation therapy (66%). Four themes were developed in the free text responses that describe problems in the utilization of occupational therapy in palliative care. Conclusions: The evaluation shows that occupational therapy in palliative care offers a variety of possible applications and approaches. The findings urgently need to be implemented in daily practice to improve the status of occupational therapists. This requires appropriate billing modalities and clear guidelines. There is still a significant need for research and training in this area.

## 1. Introduction

As their disease progresses, people with palliative illness have difficulty managing their daily lives alone and participating in activities that are meaningful to them [1]. The accompanying loss of their role can contribute to social isolation [2]. The goal of occupational therapy is to “*empower people of all ages [...] in performing activities that are meaningful to them in the areas of self-care, productivity, and leisure in their personal environment*” [3]. For palliative patients, the implementation of the individuals’ goals would mean an improvement in their quality of life by enabling them to participate in daily life or their occupation even in the last phase of life. The focus of palliative care research is mostly on medical aspects, such as pain and symptom control, and little research exists in the areas of independence, participation in life, and occupation by occupational therapists. The European Association for Palliative Care (EAPC) sees occupational therapy as an essential member in the interdisciplinary care of palliative clients [4]. Despite the high demand, occupational therapists, in contrast to physiotherapists, represent a less known professional group, and their involvement in areas of palliative care is lower [4]. This was also demonstrated using data from a cross-sectional study, which was conducted as preliminary work to the present study by our study team, in the three palliative care settings of German hospices, palliative care units, and specialized outpatient palliative care services [5]. The main reasons cited were the shortage of specialists or insufficient capacity for home visits and the lack of funding options [5]. In the 2018 study carried out by Eva and Morgan, a Europe-wide survey was conducted regarding occupational therapy care in palliative care. This has resulted in the need for further occupational therapy research and awareness of the occupational therapy role in palliative care [6]. As only five German occupational therapists participated in the survey by Eva and Morgan, no meaningful conclusions were drawn for German palliative care [6,7]. For this reason, the present cross-sectional study aims to collect a larger sample of occupational therapists working in palliative care from Germany. 

The research questions of the survey are:

- What is the involvement/integration of occupational therapy in palliative care?
- In which areas do occupational therapists work with palliative care patients?
- What specific roles do occupational therapists play in palliative care?
- What is the perceived need for research or continuing education for occupational therapists in palliative care according to these participants?

## 2. Methods

The survey of occupational therapists working in Germany was conducted with an online questionnaire via the SurveyMonkey^®^ (San Mateo, CA, USA) platform. Study design, content, and recruitment procedures, as well as data management activities, are in accordance with the ICH Guidelines of Good Clinical Practice (GCP) and were reviewed and approved by the Ethics Committee of the Department of Human Medicine at the Philipps University of Marburg (No. 33/22). The survey is registered in the German Registry of Clinical Studies (DRKS00029197). The evaluation is based on the “Checklist for Reporting Results of Internet E-Surveys (CHERRIES)”.

### 2.1. Questionnaire Design

The questionnaire is adapted from a study that was conducted in advance, as well as from the EAPC survey by Eva and Morgan from 2018 [6]. It includes 17 questions, which are divided into three topics. Part one includes general demographic questions, such as gender, age, professional qualifications, years on the job, and whether there is any collaboration with palliative clients. The last question led further to part two of the questionnaire. If the respondent states that there is no collaboration with palliative patients, it asks whether there would be a basic interest in this and what the possible reasons for a lack of collaboration are.

If, on the other hand, the respondent states that he or she works in the field of palliative care, the second part of the questionnaire focuses on this work in palliative care. The questions include the scope of employment and the specialized area in which the occupational therapy work takes place, which therapies are carried out, and how many palliative clients are worked with on average.

With the third part of the questionnaire, the respondents can describe whether occupational therapy is used in a meaningful way in palliative care and if more research in this area is considered necessary. The questionnaire concludes with a description about one’s role as an occupational therapist in palliative care.

All questions are mandatory except for free text responses. The questionnaire was discussed in advance with the working group “Palliative Care” of the German Association of Occupational Therapy, which resulted in minor adjustments. Subsequently, the questionnaire was tested by four persons working in the palliative care sector.

### 2.2. Sampling and Recruitment

Participation in the survey first assumed that the participants were at least in training/studying to become occupational therapists. The second part of the questionnaire also required that respondents work in the context of palliative care or work with palliative clients. Furthermore, only therapists who are members of the German Occupational Therapy Association (German abbreviation: DVE) were included, as the survey was conducted in cooperation with the DVE.

Data were collected over a three-month period (29 April–31 July 2022). During this period, DVE members were invited to participate in the survey in three different ways: three times via the DVE newsletter (29 April 2022, 4 June 2022, 2 July 2022), simultaneously via a study call on the DVE homepage (28 April 2022, 30 May 2022, 29 June 2022), and once via a QR code at the DVE congress on 19 May 2022. The participants were informed about the survey and the study purpose by an information letter. By voluntarily participating, they consented to study participation and anonymized data collection and processing. Participants were not offered any financial incentive for participation.

### 2.3. Data Analysis

The completed questionnaires were first checked for completeness by the study team. Duplicates were not possible due to the server settings on SurveyMonkey^®^, so a duplicate check was not necessary. The data were analyzed by means of descriptive statistics, using Microsoft Excel^®^ Version 16.6.

## 3. Results

A total of 282 responses were received, of which 281 could be included in the analysis. With the first four questions, demographic data of the participants were asked. The fifth question was a key question, as it asked whether the participant works with palliative care patients. As a result, 132 (47%) questionnaires had an automatic dropout for the main part of the survey, as the question was answered in the negative (compare the explanations of the questionnaire design above). 

**Figure 1:**
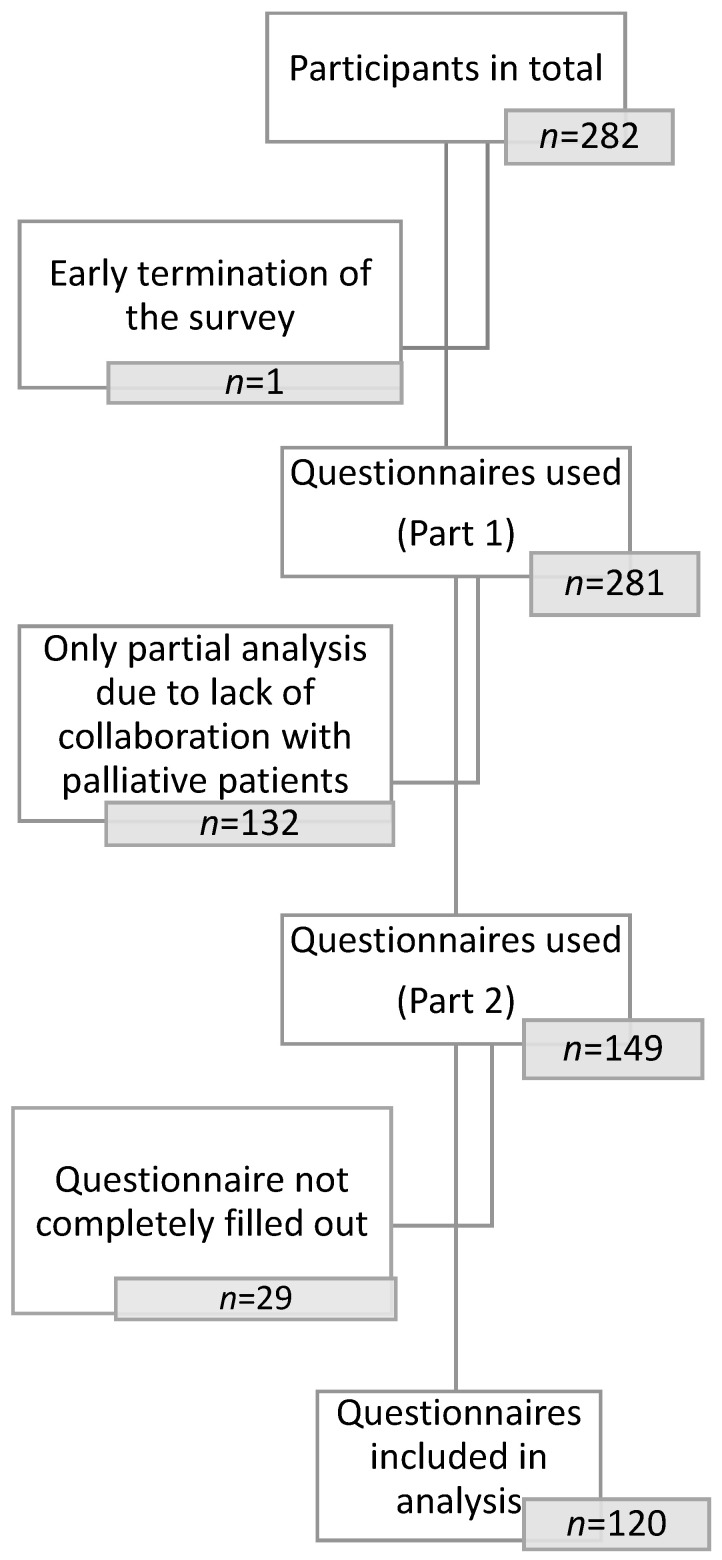
Flowchart showing the included questionnaires.

This left 149 (53%) responses from occupational therapists working in a palliative context for further analysis. Here, there were again dropouts (*n* = 29), where the questionnaire was prematurely abandoned and thus not completely filled out. A total of 120 (43%) questionnaires could be included in the detailed evaluation (Figure 1).

### 3.1. Respondent Characteristics

The demographic characteristics of all respondents are summarized in Table 1. Overall, more female occupational therapists (92%) participated in the survey. However, this high number of female participants is in line with the membership statistics of the DVE, where 85% of occupational therapists are female [8]. Most participants are between 25 and 45 years old. The majority (62%) have completed vocational training, 14% have an academic degree, and under a quarter of the participants (24%) are trainees or students.

### 3.2. Fields of Application and Tasks of Palliative Occupational Therapy

Table 2 shows the different areas of work, as well as other key figures based on the scope of work. Most participants work in several areas, with half of the participants stating that they carry out home visits in the outpatient area. Interestingly, one third work with palliative patients in the clinic outside a palliative care unit. It is also worth mentioning that occupational therapy in the hospice (4.1%) and in collaboration with specialized outpatient home palliative care (also 4.1%) make up the smallest proportion. The largest proportion (39%) of participants had worked in the field of palliative care for less than five years. In comparison, one third had been working in palliative occupational therapy for more than 10 years. In addition, 8% of the respondents work with children/adolescents and 92% with adult patients, of which, again, most work with seniors. Regarding the employment relationship, there is a relatively equal distribution between part-time (34.1%), full-time (28.3%), and self-employment (35.0%).

**Figure 2:**
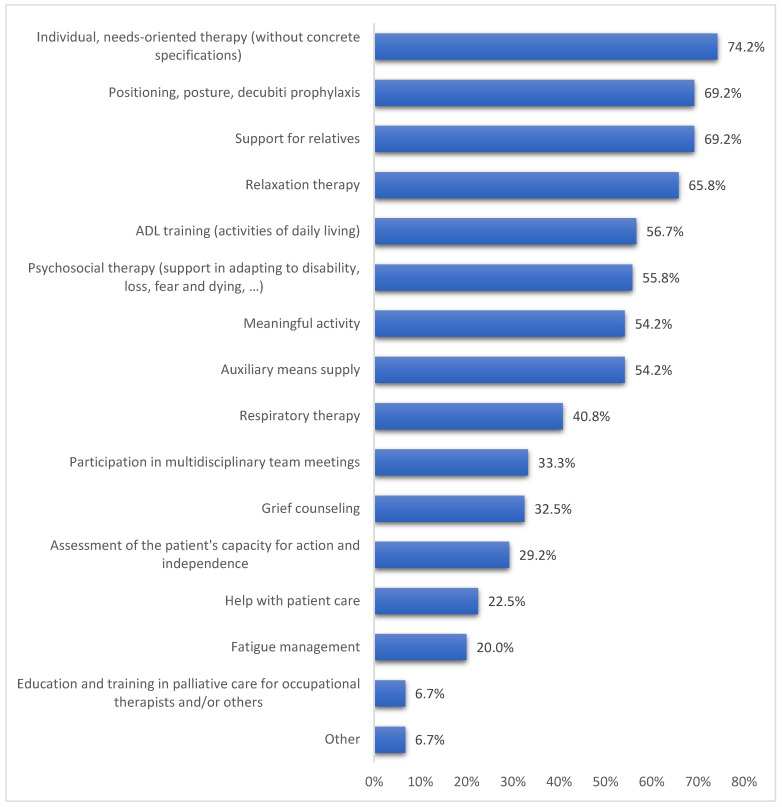
Areas of application/tasks in palliative care.

The tasks in the work with palliative patients are distributed very heterogeneously, which is shown in Figure 2. A distinction can be made between patient-related activities (e.g., breathing training or relaxation exercises) and non-patient-related activities (counselling for relatives or educational work). Most participating occupational therapists carry out patient-oriented activities, with needs-oriented therapies (without concrete specifications) being the most common (74%). Other tasks that influence the personal well-being of the clients are, overall, the focus of daily work. Support for integration into the home environment and maintaining independence through aids is also offered by more than half of the participants. Evaluating the activities that are not related to the patient, it becomes apparent that support for relatives, with 69%, takes up the largest share. After that, organizational tasks, such as participation in multidisciplinary team meetings (33.3%) or the use of assessments and evaluations, are often named. Only 7% of the participants are employed in education and training.

### 3.3. Free-Text Responses

For some dichotomous questions, the participants could additionally give free-text answers for a further explanation. Furthermore, there was a free-text question in which the participants were asked to describe their own role in palliative care. Overall, the answers were categorized in the sense of a qualitative evaluation, and four sub-themes were identified. Exemplary answers were selected and quoted for the presentation.

### 3.4. Topic I: One’s Role in Palliative Care

The respondents state that they see themselves as an important part of palliative care. They describe that it is important for the clients and their relatives to have an independent caregiver. It is noted in this context that the relatives often forget themselves. Many state that they actively promote the participation of the patients and thus maintain the dignity of the person even in the dying process.

“I think it’s important to enable quality of life through activity, regardless of the threat of end of life.”

(R-130)

“No different than in other areas of occupational therapy. We support everyday life or the management of it. We enable participation in it. Our banner says: ‘everyday life’.”

(R-238)

“Occupational therapy care is of great importance to patients. It covers many areas, involves time, can reduce fears and is of great benefit due to the professional competence. Unfortunately, it still lacks recognition by other professions or among physicians...”

(R-77)

The previous quote already addresses underrepresentation. Respondents often wish for more recognition and full integration into the team. They would like to offer more end-of-life therapy, but there is a lack of prescriptions from physicians to be able to make home visits, for example:

“It has been my experience that to some primary care physicians and specialists, the expertise of occupational therapists in palliative care is not present nor seems significant, so some clients or their families have to “fight” for prescriptions.”

(R-23)

Furthermore, the respondents describe that the accompaniment and support of clients and their relatives is a core task of palliative work. The independence and self-determination of clients should be maintained at the end of life through dignified and needs-oriented support, as well as psychoeducation and guidance of (caring) relatives. Likewise, some participants state that they see themselves as a link among patients, relatives, and caregivers. Overall, however, palliative occupational therapy is also described as demanding, very comprehensive, creative, and sensitive.

“I try to do everything within my power as a therapist to help the patient do as much as I can. Whether it’s going for walks, cooking, baking, playing games, laughing, reminiscing, massage, relaxation exercises or movement exercises. The tasks are as varied and different as each patient is different.”

(R-51)

### 3.5. Topic II: Stressful Situations

Another question deals with whether working with palliative patients is perceived as stressful. Out of 119 answers, 68 (57%) of the respondents’ state that this is the case. Those who find the work stressful cite time pressure, structural requirements, emotional strain, a lack of exchange, and helplessness, especially when patients are in severe pain, as key issues.

“Related to ignorance among other health care professionals: The constant justification for the work done as well as explanation of the profession. The term “meaningful” is very vague and not certainly tangible to others. This weighs on me. Regarding palliative work with clients, unfortunately we are often only called in at a very late stage, although we could already make a valuable contribution to the participation of the clients at an earlier stage. This is often very frustrating and also stressful when you know that you could have enabled more participation with quite simple redesigns/adaptations of the environment.”

(R-281)

### 3.6. Topic III: Utilization of Occupational Therapy Expertise

The previous quotation can also be used for the topic of occupational therapy utilization. Out of 119 participants, 65 (55%) are of the opinion that expertise is used correctly, i.e., in line with the profile of an occupational therapist. In contrast, 46 (39%) of the respondents denied this, often citing “ignorance” as the main reason:

“Ignorance regarding the variety of tasks of occupational therapy often hinders its adequate use in palliative care. For example, it is often assumed that occupational therapists can only ‘tinker’ and have no expertise in caring for palliative clientele. Facilitating meaningful activity is a tenet of occupational therapy that is elusive for many. Because of this, we often experience a fundamental problem with our profession, that no one really knows what occupational therapists actually do. Occupational therapy expertise is a very suitable therapy method in palliative care by enabling participation until death—it is just unfortunately considered far too rarely.”

(R-281)

The lack of interdisciplinary exchange or collaboration is also mentioned several times, especially in relation to assistive technology care:

“Clearly that would be the responsibility of the medical supply stores, but so far I haven’t found any that would do extensive consultation. I see us as coordinators who would have to build a network or fit into a network so that optimal care for the patient takes place.”

(R-254)

Meanwhile, this is often cited as ignorance of the occupational therapy task variety among health care workers:

“The world doesn’t know about our expertise. Neither doctors nor nurses. Maybe even we ourselves don’t.”

(R-238)

“It needs a clearly formulated and delimited task, which corresponds to the occupational therapy and is so also mediatable.”

(R-77)

These quotes also show that occupational therapists themselves may not be able to precisely define their work assignment in palliative care and are entering uncharted territory when working with palliative patients. This may also be due to the lack of education and training opportunities in this area.

“More intensive attention to this area already in training. Continuing education opportunities.”

(R-31)

### 3.7. Topic IV: Opportunities for Improvement

Furthermore, the interviewees have been asked about possible improvements for the work and acceptance of occupational therapists in palliative care.

“Occupational therapy is often not considered in the provision of care. Getting us on board early on, we could accomplish even more....”

(R-213)

In this area, as well, the lack of awareness of occupational therapy is cited as the main issue. Structural requirements are another main topic. The respondents would like the topic of palliative care to be firmly anchored in training courses and not just touched upon, and likewise, better integration in guidelines, as well as funding possibilities by health insurance companies. Furthermore, close cooperation with regional palliative networks and outpatient palliative services is suggested.

“As well as basically occupational therapy is not self-explanatory, in palliative care is a lot of knowledge transfer about occupational therapy necessary. Public relations work, participation in the DGP, networking with the regional hospices/palliative care units, updating the existing information material of the DVE”

(R-243)

### 3.8. What Occupational Therapists Who Do Not Work in Palliative Care Think about Palliative Work

The 132 respondents who stated that they do not work with clients in a palliative phase of life were asked whether they would like to do so. Here, 75% state that they would like to work in a palliative context. Reasons for not working in the palliative context are e.g., a lack of further training or the fear of not getting support in the team or no supervision in case of stress. The statement that physicians stop prescribing occupational therapy as soon as a patient is transferred to palliative care also shows that occupational therapists are deprived of the opportunity to work in the palliative setting.

On the other hand, 20% state they do not want to work in the palliative context. The reasons given for this are their own preoccupation with the issues of dying, death, and mourning and fear of the emotional burden. Some of the respondents’ state that they would prefer to work in another field.

Occupational therapists who already work in palliative care were asked if they would like to work more in this field. Of the respondents, 62 (52%) answer that they would like to work more in the palliative context; for 16 (13%) respondents, the amount of work is fine that way, and 42 (35%) are unsure.

In addition, 81% of the respondents would like to see more research in palliative occupational therapy. However, only 28% state that they would like to engage in research themselves, 42% are unsure, and 31% do not want to do research.

## 4. Discussion

This study provides information about the work of occupational therapists in the field of palliative care in Germany and highlights the exact fields of activity in this context and what problems exist in the applicability. Compared to the European study by Eva and Morgan from 2018, in which only five German occupational therapists participated, a more detailed picture of the German care can be presented with the present survey [6].

### 4.1. Significance of the Findings

Therapy at the end of life means, for the participating occupational therapists, that meaningful activity should be made possible until the end of life. As the main task of occupational therapies in palliative care, the respondents state needs-oriented therapy according to the patients’ needs, as well as the provision of aids, family support, relaxation therapies, and positioning. At the same time, many express frustrations that their role is often “limited” to a few areas of application and that their occupational expertise is not seen in the palliative setting. These findings are also found in Eva and Morgan’s European survey, as well as in American and Australian studies [6,9,10,11,12].

When looking at the areas of responsibility of occupational therapists, slight differences can be seen between the present survey and the study by Eva and Morgan. Whereas the cohort of German occupational therapists mainly indicates needs-oriented therapy according to the patient’s requirements and caring for relatives as the most frequent tasks, in the European comparison, the provision of auxiliaries, assessing function, and independence, as well as training for relatives and colleagues, are more important. In Germany, participation in interdisciplinary meetings or work in education seems to play a rather subordinate role in daily work.

However, the relevance of needs-oriented therapy, which is stated as the most common form of therapy, is also reflected in the role descriptions of occupational therapy associations in Canada, America, and Germany: “*Client-centered occupational therapy is oriented towards the complex needs of the ill person. These include not only the physical needs, but also the social, psychological, emotional and spiritual needs of the affected persons and their relatives. Occupational therapists thus help to preserve autonomy and quality of life*” [13,14,15,16]. This approach allows the needs of clients and their families to be identified and prioritized in relation to the last phase of life [12]. According to Aitken, the need to be actively involved in everyday life remains despite the disease [17]. In order to meet this need, occupational therapy can contribute by providing aids and adapting to the environment [17]. Melanie Blichfeldt and colleagues have confirmed this in their survey of cancer patients. They suggest that activities of daily living and social interactions are of particular importance in treatment [18].

Of those surveyed, 57% stated that they frequently experience stressful situations. Above all, time pressure, structural requirements, mental stress, a lack of exchange, and helplessness were named as stressful factors. Also, 20% of those who do not work in palliative care stated that dealing with the issues of death and dying would be an obstacle for them to work in this field. As early as 1995, Mary Vachon was able to show in her review that employees in the field of palliative care are generally less affected by stress and burnout than professionals in other medical fields [19]. Dealing with death and dying is often stressful for staff outside palliative care, so it is not surprising that 20% state that they would not want to work in this field. By contrast, medical professionals in palliative care perceive organizational and social problems as stressful, especially when emotional support is lacking or the workload is too high [19]. Thus, our results are consistent with the problems reported in the literature.

In this survey, a lack of knowledge of other medical professionals about the scope of the occupational therapy role is identified as a barrier. Overall, 39% report that they believe occupational therapy expertise is not properly utilized. Georgia Halkett and colleagues found similar results in their qualitative survey of Australian occupational therapists and health professionals. They were able to identify four barriers that impede occupational therapy treatment in palliative care [9]. These include the contribution occupational therapists can make in palliative care, insufficient knowledge about treatment options, inadequate funding opportunities, and limited research in palliative occupational therapy [9]. Chow et al. also demonstrate the same problems in their survey of U.S. occupational therapists and policy makers [20]. In the institutional survey of German hospices, palliative care units, and specialized outpatient palliative care services conducted by our research group in advance of the present survey, we also found that occupational therapy is not used as frequently as physiotherapy [5]. Particularly in home-based outpatient care, there are large deficits in the range of services offered, which is often justified by financing problems, although occupational therapy is a cost-effective form of treatment due to its preventive character and because higher follow-up costs can be avoided [5,21]. This dissatisfaction, especially with regard to adequate funding for occupational therapy, is also reflected in the survey of occupational therapists presented here but seems to be not only a German problem [9,20]. It is often noted that the supply is too low and occupational therapy is too rarely prescribed by physicians.

Education and training are frequently mentioned topics in the free-text responses, both among occupational therapists who work in palliative care and among those who indicated that they do not work in this field. Continuing education should be offered in both directions. On the one hand, it is about training other professional groups about the possibilities of occupational therapy care, but also about content for occupational therapists about possibilities of palliative care for their patients. The latter is already demanded by many for the training of occupational therapists. This is also evident, for example, in the survey by Georgia Halkett, who states that research should be conducted on the role, contribution, and benefits of occupational therapy in palliative care [9]. Eva and Morgan have also elaborated that in addition to a lack of research and evidence for occupational therapy, a lack of educational opportunities in Europe is also a problem [6]. Occupational therapy is often associated with rehabilitation, so an understanding of its use in palliative care is lacking [20]. The scoping review by Chow and Pickens concludes that evidence already exists regarding the effectiveness of general occupational therapy interventions and end-of-life interventions, but not regarding specific occupational therapy interventions, such as environmental adaptations or meaningful activity [22]. They believe that empirical research will validate the effectiveness of occupational therapy, justify its use and funding, and improve access to activity-based interventions for people at the end of life [22]. These study results thus suggest that our findings thus do not represent a specific problem in German palliative care, but that occupational therapy treatment is inadequately implemented in palliative care in many countries.

### 4.2. Limitations of This Study

Certain factors must be considered for the interpretation of the evaluation. The invitation to participate in the survey only reached occupational therapists who are members of the German Occupational Therapy Association. This means that not all occupational therapists in Germany were reached, so it is not easy to generalize the results. In addition, the survey was an anonymous online survey. Especially, the free fields were not always filled out, because in the questionnaire, the questions could partly be skipped. Even if the survey was anonymized, a bias towards socially desirable answers is possible.

## 5. Conclusions

The needs-oriented support of seriously ill people and the accompanying enabling of participation in everyday life until the end of life show how important occupational therapy work is in palliative care. However, there are also many occupational therapists who feel that this potential is not fully seen by other health care professions. For example, there is often a lack of opportunities for home visits to promote participation in normal daily life. Therefore, more educational work should be performed on the professional expertise and possibilities of palliative occupational therapy to adequately support patients in their sphere of activity. In this context, the training of occupational therapists should also focus more on the topic of palliative care, as well as collaboration on guidelines to manifest occupational therapy in palliative care.

Further research in the context of palliative occupational therapy should be conducted to further evaluate the professional expertise and to be able to scientifically substantiate the positive aspects of occupational therapy interventions. Furthermore, comparisons to other professions should be made to identify commonalities for interdisciplinary care.

## Figures and Tables

**Table 1:** Characteristics of the respondents.

		*n* = 281	Percentage
Gender	Female	258	92.1
	Male	19	6.7
	Divers	3	1.0
Age (years)	<25	51	18.1
	25–35	78	27.7
	36–45	66	23.4
	46–55	53	18.5
	>55	34	12.1
Qualifications	Currently in training/studying	67	23.9
	Completed vocational training	174	62.1
	Bachelor’s degree	32	11.4
	Master’s degree	9	3.2
	Others	16	5.7
Working with Palliative clients	Yes	149	53.0
	No	132	47.0

**Table 2:** Distribution of palliative work.

		*n* = 120	Percentage
Number of years working with palliative clients	<5	47	39.1
	6–10	37	30.8
	11–20	31	25.8
	21–30	4	3.3
	>30	1	0.8
Primary collaboration	Children/adolescents	10	8.3
	Adults	62	51.6
	Older people (>65 years)	94	78.3
Employment relationship	Part-time employed	41	34.1
	Full-time employed	34	28.3
	Self-employed	42	35.0
	Voluntary work	6	5.0
	Other	5	4.1
Working area	Clinic (outside a palliative care unit)	40	33.3
	Palliative care unit	24	20.0
	Hospice	5	4.1
	Specialized outpatient palliative care team	5	4.1
	Medical practice	46	38.3
	Home visits (outside specialized palliative care)	60	50.0

## Data Availability

Data are available on request. A request can be made to the corresponding author.
